# Isolated low-grade serous carcinoma arising in inguinal lymph nodes in the setting of endosalpingiosis: A case report

**DOI:** 10.1016/j.gore.2025.101769

**Published:** 2025-05-23

**Authors:** Samantha A. Solaru, Marisa C. Liu, Vincent Lee, Robert E. Bristow

**Affiliations:** a Department of Obstetrics and Gynecology, University of California, Irvine-Medical Center, 3800 West Chapman Avenue, Suite 3400, Orange, CA 92868, USA; b Department of Gynecologic Oncology, University of California, Irvine-Medical Center, 3800 West Chapman Avenue, Suite 3400, Orange, CA 92868, USA; c Department of Pathology, University of California, Irvine-Medical Center, 3800 West Chapman Avenue, Orange, CA 92868, USA

## Abstract

**Introduction:**

Low-grade serous carcinoma (LGSC) is a rare, indolent subtype of epithelial ovarian cancer that often arises from precursor lesions in the ovary or peritoneum and is associated with MAPK pathway mutations. Unlike high-grade serous carcinoma (HGSC), which typically originates from cells in the fallopian tube, LGSC shows limited response to chemotherapy. An isolated presentation in an extraperitoneal site, such as an inguinal lymph node, is exceedingly rare. We present a case of primary nodal LGSC arising in the setting of endosalpingiosis.

**Case:**

An 80-year-old woman presented with a two-year history of left lower quadrant pain and a newly enlarging left groin mass. Imaging identified a vascular left inguinal mass, and initial biopsy favored HGSC. Staging procedures including hysteroscopy, dilation and curettage, and diagnostic laparoscopy with bilateral salpingo-oophorectomy showed normal pelvic organs with no evidence of malignancy. Chemotherapy was initiated but resulted in only a modest response. Surgical resection of the mass itself revealed LGSC with adjacent endosalpingiosis. With no evidence of primary disease elsewhere, a diagnosis of primary inguinal node LGSC arising from endosalpingiosis was made.

**Conclusion:**

This case highlights the diagnostic challenges of isolated LGSC without a detectable primary site. Initial misclassification can lead to suboptimal management. Accurate diagnosis requires thorough surgical and pathological evaluation to ensure appropriate treatment in these rare and atypical presentations.

## Highlights

• Low-grade serous carcinoma (LGSC) can arise in isolated extra-ovarian locations, including inguinal lymph nodes.
• Accurate histopathologic diagnosis, including IHC and molecular profiling, is critical in distinguishing LGSC from HGSC.
• Misclassification of serous carcinomas can lead to inappropriate treatment strategies and delayed definitive management.
• Recognition of endosalpingiosis and its potential malignant transformation is important in evaluating gynecologic lesions.
• Treatment of isolated extra-pelvic LGSC lesions should mirror that of more common adnexal disease.

## 1 Introduction

Tubo-ovarian serous carcinomas have been graded using a multitude of systems including the FIGO system, the WHO system, the GOG system, and many others. In 2014, Kurman et al proposed a two-tiered system, dividing serous tumor types into low-grade and high-grade, according to differences in biological behaviors, response to therapies, and prognosis (Köbel and Kang, 2022, Kurman et al., 2014). Low-grade serous cancer (LGSC) is a rare type of cancer thought to arise from inclusion glands within the ovary and can resemble normal ovarian tissue. This cancer is typically characterized by slow progression, a low mitotic index, and architecture that typically involves micropapillae. Mitogen-activated protein kinase (MAPK) alteration including KRAS and BRAF mutations are prominent in up to 50 % of LGSC tumors (Grisham et al., 2023). While the prognosis of LGSC compared to high-grade serous carcinoma (HGSC) is typically better, the response to chemotherapy is generally poor with studies reporting response rates as low as 5 % in the recurrent setting (Gershenson et al., 2009). Alternatively, HGSC tumors have a high mitotic index, are clinically more aggressive, and carry a poor prognosis. They are widely thought to arise from the epithelium of ovary and fallopian tube (Vang et al., 2013). These tumors will typically carry a P53 mutation and have defects in the pathways that contribute to DNA repair. The current report describes a unique case of primary inguinal lymph node LGSC arising in the setting of extraperitoneal endosalpingiosis, a typically benign condition where glandular tissue resembling the fallopian tubes is seen in abnormal locations.

## 2 Case Description

An 80-year-old woman with a remote past surgical history of ovarian cyst removal presented with complaints of two years of left lower quadrant pain and one month of left groin swelling and an inguinal mass. She maintained a very active lifestyle as a belly-dancing instructor and was in excellent physical health.

**Fig. 1:**
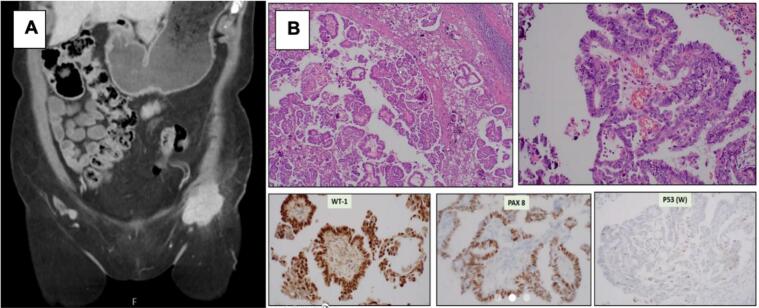
(A) CT Abdomen and pelvis, 4.8 × 4.6 × 4.8 cm left inguinal mass (B) Histology and IHC staining of core needle biopsy. WT-1 positive, Pax 8 positive, P53 wild-type.

On initial presentation to the patient’s primary care provider, the inguinal mass was palpable. Ultrasound imaging revealed a 5.6 cm vascular left inguinal mass that was concerning for abnormal lymphadenopathy. Subsequent CT imaging showed a solid 5.2 ×  4.1 ×  3.4 cm lesion in the left hemi-vulva with thickening of the lower vaginal wall and a partially necrotic 4.8 ×  4.6 ×  4.8 cm left inguinal mass (Fig. 1A). An IR-guided biopsy and fine-needle aspiration of the inguinal mass revealed metastatic serous carcinoma, favoring ovarian origin with IHC staining PAX-8 positive, WT-1 positive, and p53 wild-type, Ki67 staining was not performed (Fig. 1B). The patient was referred to Gynecologic Oncology.

In an effort to establish the site of origin (ovaries, fallopian tubes, endometrium), the patient underwent a hysteroscopy, dilation and curettage, as well as diagnostic laparoscopy with bilateral salpingo-oophorectomy. Operative findings included normal appearing tubes and ovaries bilaterally. The uterine cavity was noted to have several filmy fragments reminiscent of cystic change without a gross mass. No vaginal findings were appreciated as previously seen on imaging. Final pathology returned without significant histopathological changes and there was no trace of malignant tissue. The tissue samples were also provided to the Johns Hopkins Surgical Pathology Consult Service who agreed with the previous diagnosis, no evidence of malignancy was identified. Given no evidence of primary malignancy was identified in the uterus, fallopian tubes, or ovaries, it was thought that this was likely a high-grade serous carcinoma or unknown origin or originating from endosalpingiosis. Based on clinical exam and imaging, the left inguinal tumor mass encased the left femoral artery and vein; therefore, initial management with neoadjuvant chemotherapy with carboplatin and paclitaxel (CBCDP (AUC5) / PAX (135 mg/m2)) was initiated with the goal of achieving a partial clinical response and converting the patient into a surgical candidate.

**Fig. 2:**
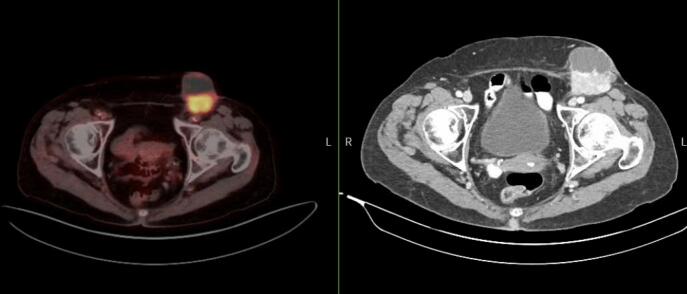
(left) PET-CT scan, Left inguinal nodal mass with solid portion significantly FDG avid consistent with neoplasm. No additional abnormal uptake in the abdomen/pelvis. (right) CT Abdomen and Pelvis, Large, solid, and cystic left inguinal nodal mass with extension into the underlying abdominal wall musculature. This measures about 5.6 × 5.3 cm (previously 4.8 ×  4.6 cm).

After five cycles of neoadjuvant chemotherapy with carboplatin and paclitaxel, the patient’s CA 125 decreased from 295 U/ml to 78 U/ml. A surveillance PET-CT scan obtained demonstrated a modest interval decrease in size with regression from the femoral vasculature (Fig. 2).

**Fig. 3:**
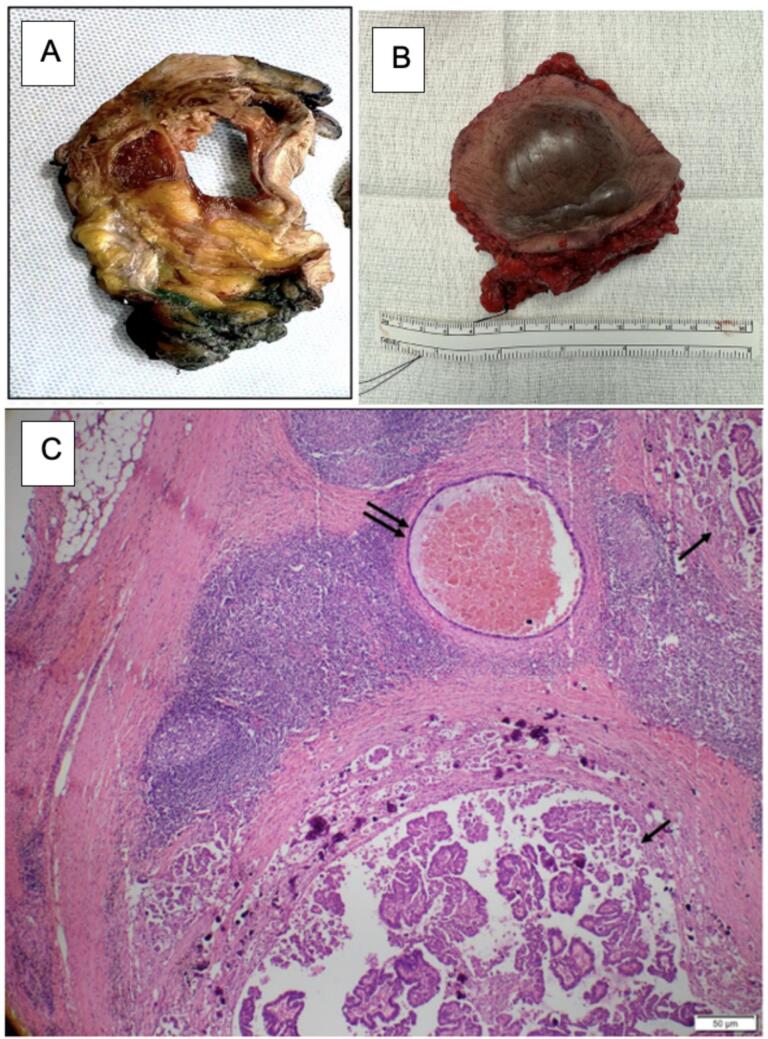
(A) Gross specimen, 8.5 ×  7.8 ×  4.5 cm piece of soft tissue with overlying skin, sliced. (B) Gross specimen resected soft tissue with overlying skin. (C) Low-power view of the left inguinal lymph node involved by low-grade serous carcinoma with mild nuclear atypia and papillary architecture (single arrows), with adjacent endosalpingiosis composed of cystically dilated gland lined by a single layer of bland ciliated epithelium (double arrows).

Six months after initial diagnosis, the patient then underwent an en bloc resection of left inguinal tumor mass resection with associated lymph nodes, overlying skin, the left inguinal ligament, and residual left round ligament stump in a combined case with Gynecologic Oncology, Vascular Surgery, and Plastic Surgery. Grossly, the specimen was an 8.5 ×  7.8 ×  4.5 cm piece of soft tissue with overlying skin (Figs. 3A and B). Histological sections showed a tumor with final diagnosis of low-grade papillary serous carcinoma involving the lymph nodes with negative margins and adjacent endosalpingiosis (Fig. 3C). IHC staining showed the tumor was positive for WT-1, PAX-8, p53 (wild type), P16 (patchy), and ER while negative for both HER2 and PR with a low proliferative index (<5%) on Ki-67. In the absence of tumor in both the bilateral fallopian tubes and ovaries as well as the absence of peritoneal disease at the time of diagnostic laparoscopy, the diagnosis of primary inguinal nodal LGSC from endosalpingiosis was made.

## 3 Discussion

Endosalpingiosis is the presence of ectopic cells that are both structurally and functionally similar to those seen in the normal fallopian tube epithelium. The pathogenesis is similar to that of more commonly seen and studied endometriosis and the two processes together are described in the literature as Mullerianosis. As endosalpingiosis contains tubal-like epithelium, it is not known to cause an inflammatory response as does its endometriosis counterpart, so the diagnosis is often limited to the surgical removal of tissue for another benign gynecologic issue. A study by Sunde et al evaluated 1,148 patients with gynecologic specimens collected for various reasons, such as abnormal uterine bleeding, sterilization, risk-reducing surgery, or malignancy. All submitted specimens with benign-appearing adnexal masses were reviewed and found that endosalpingiosis was present in 22 % of all cases (p < 0.001), with a significant increase in prevalence among postmenopausal women (up to 66 %). These findings suggest that endosalpingiosis is more common than previously thought, particularly among older women (Sunde et al., 2020). Some imaging findings consistent with endosalpingiosis have been reported, with ultrasounds demonstrating expansile hyperechoic or anechoic masses and CT findings typically demonstrating well circumscribed masses with both cystic and solid components as is the case with this patient (Katre et al., 2010). MRI findings are not as well described in the literature though one case report documents a hyperintense T2 and hypointense T1 signal (Fujii et al., 2021). A systematic review by Burla et al set out to describe the intraoperative findings of endosalpingiosis. They reported that the vast majority of lesions consistent with endosalpingiosis were only millimeters in size, vesicular in presentation, and most frequently were found along the uterosacral ligaments and the pouch of Douglas (Burla et al., 2022).

Recent studies have identified a relationship between endosalpingiosis and gynecologic tumors. A retrospective case-control analysis of 967 patients with a histologic diagnosis of either endometriosis or endosalpingiosis at an academic center over a 20-year period found that endosalpingiosis was present concurrently with malignancy in 40 % of specimens across all subgroup analyses (Lewis et al., 2022). There have been a few cases reported of endosalpingiosis occurring concurrently with ovarian cystadenomas in a pattern that mimics metastatic ovarian cancer (Cox et al., 2020, Singhania et al., 2014). These cases cited the importance of obtaining tissue samples to utilize IHC staining and molecular studies to be able to arrive at the proper diagnosis so as to avoid over or under-diagnosing malignancy.

Typically, p53 IHC staining wild-type or null type is consistent with a low-grade serous carcinoma. In this particular case, this patient’s initial tumor sample from a core biopsy exhibited p53 wild type staining though was interpreted as a high-grade serous carcinoma; however, the diagnosis was later modified to low grade serous carcinoma after complete resection. It was because of the initial diagnosis that this patient received 5 cycles of neoadjuvant chemotherapy, and likely accounts for the only modest clinical response. Tumor p53 gene variants are typically a reliable way to distinguish HGSC from LGSC however, it has been reported that up to 4 % of high-grade serous carcinomas can be p53 variant negative. Kasherman et al describes two cases of stage III HGSC with wild type p53 staining, expression of WT-1, and PAX-8, even in the setting of a known TP53 mutation (Kasherman et al., 2021).

There have been three cases previously reported in the literature of isolated inguinal node LGSC (Sah et al., 2020, Carrick et al., 2003) with the presence of findings consistent with associated endosalpingiosis. There are even fewer reported cases of isolated inguinal node HGSC with most cases being identified as a recurrence of disease rather than an isolated pathology. Appropriate sampling and IHC studies should be used to aid in diagnosis. Obtaining the correct diagnosis after initial workup is paramount to avoiding delays in initiation of appropriate care.

In this case, the patient underwent chemotherapy based on an initial assumption of an HGSC diagnosis but demonstrated a suboptimal response, aligning with the final diagnosis of LGSC which again was confirmed upon reexamination of the initial tissue specimen. Consequently, institution of definitive treatment, consisting of surgical resection and hormonal adjuvant therapy, was delayed.

The current case report underscores the vital role of expert pathology interpretation in ensuring a timely and accurate diagnosis to guide appropriate management of extra-ovarian LGSC. While discrepancies in pathology findings may raise some suspicion for synchronous LGSC and HGSC, it is important to note that these cases are exceedingly rare and not well described in the literature. Clinicians should maintain a high index of suspicion for an endosalpingiosis origin when an isolated LGSC lesion is detected without a clear primary site in the uterus, fallopian tubes, or ovaries. In such cases, treatment should align with the standard management of this disease as it typically presents in the pelvic organs, even in the setting of local advancement.

**Written informed consent was obtained from the patient for publication of this case report and accompanying images. A copy of the written consent is available for review by the Editor-in-Chief of this journal on request.**

## CRediT authorship contribution statement

**Samantha A. Solaru:** Writing – original draft, Writing – review & editing, Investigation, Data curation. **Marisa C. Liu:** Data curation. **Vincent Lee:** Data curation. **Robert E. Bristow:** Writing – review & editing, Validation, Supervision, Conceptualization.

## Declaration of competing interest

The authors declare that they have no known competing financial interests or personal relationships that could have appeared to influence the work reported in this paper.
